# Striatal Pre-Enkephalin Overexpression Improves Huntington’s Disease Symptoms in the R6/2 Mouse Model of Huntington’s Disease

**DOI:** 10.1371/journal.pone.0075099

**Published:** 2013-09-11

**Authors:** Stéphanie Bissonnette, Mylène Vaillancourt, Sébastien S. Hébert, Guy Drolet, Pershia Samadi

**Affiliations:** 1 Axe Neurosciences, Centre de recherche du CHU de Québec, CHUL, Québec, Canada; 2 Département de psychiatrie et de neurosciences, Université Laval, Québec, Canada; Hertie Institute for Clinical Brain Research and German Center for Neurodegenerative Diseases, Germany

## Abstract

The reduction of pre-enkephalin (pENK) mRNA expression might be an early sign of striatal neuronal dysfunction in Huntington’s disease (HD), due to mutated huntingtin protein. Indeed, striatopallidal (pENK-containing) neurodegeneration occurs at earlier stage of the disease, compare to the loss of striatonigral neurons. However, no data are available about the functional role of striatal pENK in HD. According to the neuroprotective properties of opioids that have been recognized recently, the objective of this study was to investigate whether striatal overexpression of pENK at early stage of HD can improve motor dysfunction, and/or reduce striatal neuronal loss in the R6/2 transgenic mouse model of HD. To achieve this goal recombinant adeno-associated-virus (rAAV2)-containing green fluorescence protein (GFP)-pENK was injected bilaterally in the striatum of R6/2 mice at 5 weeks old to overexpress opioid peptide pENK. Striatal injection of rAAV2-GFP was used as a control. Different behavioral tests were carried out before and/or after striatal injections of rAAV2. The animals were euthanized at 10 weeks old. Our results demonstrate that striatal overexpression of pENK had beneficial effects on behavioral symptoms of HD in R6/2 by: delaying the onset of decline in muscular force; reduction of clasping; improvement of fast motor activity, short-term memory and recognition; as well as normalization of anxiety-like behavior. The improvement of behavioral dysfunction in R6/2 mice having received rAAV2-GFP-pENK associated with upregulation of striatal pENK mRNA; the increased level of enkephalin peptide in the striatum, globus pallidus and substantia nigra; as well as the slight increase in the number of striatal neurons compared with other groups of R6/2. Accordingly, we suggest that at early stage of HD upregulation of striatal enkephalin might play a key role at attenuating illness symptoms.

## Introduction

Huntington’s disease (HD) is a dominant inherited neurodegenerative disease characterized by motor, cognitive and psychiatric symptoms including depression, weight loss and dementia. The disease is caused by a CAG trinucleotide expansion in the exon 1 of the huntingtin gene, which is translated into polyglutamine in the N-terminal region of HD protein [1], [2]. When the number of CAG repeats is more than 36, mutant huntingtin aggregates in the nuclei and can disrupt transcriptional factors leading to neurodegeneration [3]. Although the mutated huntingtin protein is expressed ubiquitously throughout the brain, the most striking neurodegenerative changes are first observed preferentially in striatal medium spiny neurons [4]–[6]. However, the reason of this early vulnerability is not yet well known.

The opioid system which is directly involved in many physiological effects, such as analgesia, reward, learning, memory and mood [7] is mainly present in the basal ganglia. The striatum, the input structure of the basal ganglia, and the site of interaction between dopamine (DA) and glutamate, is among the brain regions with the highest levels of opioid receptors (µ, δ, κ) and opioid peptides pre-enkephalin (pENK) and pre-dynorphin (pDYN), the precursors of enkephalin and dynorphin, respectively [8], [9]. Evidence gathered from neurochemical and pharmacological studies point to an important role of opioid peptides in the balanced and/or coordinated activity of the striatal output pathways in pathological conditions such as Parkinson’s disease [10]–[13]. Moreover, the neuroprotective properties of opioids have been recognized recently [14]. Activation of δ opioid receptors (δORs) has been shown to have neuroprotective effect against cerebral ischemia in rats [15]–[18]. In addition, opioid-mediated signaling is implicated in cell survival [19]–[21], and in protection of motor networks during perinatal ischemia [22]. *In vitro* and *in vivo* enhanced survival of DAergic neurons after neurotoxin exposure [21], and even neuroprotection against mitochondrial respiratory chain injury [23] have also been demonstrated. Other studies provided evidence of higher survival of intrastriatal grafted DAergic neurons treated with an enkephalin analog in a rodent model of PD [24].

Interestingly, an early sign of neuronal dysfunction in HD is the reduction of pENK mRNA expression due to mutated huntingtin protein [25]–[27]. Indeed, GABAergic striatopallidal (pENK-containing) neurons are more vulnerable to neurodegeneration and their loss has been seen at earlier stage of disease, even at presymptomatic stage, compared to the loss of striatonigral (pDYN-containing) neurons [25]–[27]. The pENK mRNA expression is reduced in surviving neurons at presymptomatic stage of HD [26]–[28]. However, no data are available about the role of striatal pENK in the basal ganglia motor circuit in HD. The objective of our investigation was to identify whether striatal pENK up-regulation can improve behavioral dysfunction in transgenic mice model of HD, and/or reduce or delay striatal neuronal loss. Among the transgenic mouse models, the R6/2 line is considered as a mainstay of HD research because of its rapid and reproducible progression of HD-like symptomatology including: progressive striatal neuronal loss; decline in weight gain and muscular force from 9 weeks of age; onset of abnormal dystonic limb movement (clasping) at 4 weeks; reduction in fast activity as early as 6 weeks indicating that movement speed appears to be a more sensitive measure of decline in locomotor activity compared with distance traveled alone; deficits in motor performance and coordination as early as 6 weeks; and cognitive decline [29]–[31]. In this study, we used *in vivo* novel technology using viral vector gene transfer to overexpress pENK in striatal neurons of the transgenic R6/2 mouse model of HD. Our results provide the first evidence that striatal overexpression of pENK has beneficial effects in the improvement of behavioral dysfunction in R6/2 mouse model of HD, as manifested by delay in decline of limb muscular force, reduction of abnormal clasping movement, increase of fast motor activity, normalization of anxiety-like behavior, and alleviation of different cognitive performances. The observed behavioral improvement has not been accompanied by the rescue of decline in striatal volume. However, a slight improvement of striatal neuronal number was observed five weeks following pENK gene delivery compared with other groups of R6/2.

## Materials and Methods

### Viral vector construction

The construction of rAAV2-GFP-pENK and rAAV2-GFP (control) plasmids, and the generation of adeno-associated viruses (AAVs, serotype 2/2), was carried out at Gene Transfer Vector Core, University of Iowa (www.uiowa.edu/gene/). Briefly, the full-length cDNA sequence of rat pENK (Y07503) was subcloned into the *ClaI* and *XhoI* sites of the pFBAAVmU6mcsIRESeGFP plasmid. Positive clones were verified by sequencing, and pENK (and eGFP) expression was validated in cells. The virus was then generated and purified as previously described [32], [33].

### Animals

The experiments were performed using male mutant gene carrier (R6/2) and wild type (WT) mice from the same breeding colony (B6CBATg(HDexon1)62Gpb/3J) maintained at the animal care facility in accordance with the standards of the Canadian Council on Animal Care. The breeders, males and ovary-transplanted females, were from the same strain, obtained from a line maintained at The Jackson Laboratory (Bar Harbor, ME, USA) involving a C57BL/6 and CBA background. The genotype and CAG repeat lengths expressed by offspring was determined by Laragen (Culver City, CA, USA). The number of CAG repeats in transgenic mice was 115–125. All animal experiments were accomplished under the guidelines of the Canadian Guide for the Care and Use of Laboratory Animals, and all procedures were approved by the Institutional Animal Care Committee of Laval University. Mice were weighed once a week. All behavioral protocols were performed during the light phase at the same time of the day.

### Experimental design

The study involved a wild type (WT) group and R6/2 littermates carrying mutant huntingtin protein. The latter were randomized into 3 groups: Intact R6/2 mice which received no treatment, R6/2 mice having received striatal rAAV2-GFP (GFP), or rAAV2-GFP-pENK. All striatal injections were performed at 5 weeks of age. The mice were euthanized at 10 weeks and the brains were processed for post-mortem experiments.

### Stereotaxic injection of adeno-associated virus

Following anesthesia with ketamine (100 mg/kg, intraperitoneally; ip) and xylazine (10-mg/kg, ip), the head of 5 weeks old R6/2 mice was fixed in a stereotaxic frame (Kopf Instruments, CA, USA). Bilateral injection of rAAV2-GFP as a control or rAAV2-GFP-pENK was made directly into the striatum at coordinate relative to bregma: anterior/posterior 0.62 mm, lateral/medial 1.8 mm, and 3.5 mm ventral to the dura mater [34]. Microinjections were performed using a 5 µl Hamilton syringe with a 30 gauge bevel needle. A total injection volume of 3 µl was delivered to the striatum in each hemisphere with an infusion rate of 0.1 µl/min. After injection, the needle remained in place for an additional 10 minutes to allow the diffusion of rAAV2- vectors and then it was slowly withdrawn.

### Behavioral characterization

#### Grip Strength

The muscular force of experimental mice was weekly evaluated using the Grip Strength from week 4 of age. The apparatus (Columbus Instruments, Columbus, OH, USA) can measure the peak tension (T-PK) exerted instinctively by a mouse as the examiner attempts to pull it off from a wire mesh grid by gently dragging at the base of the tail [30]. The device provides a digital readout of maximal force generated, expressed in grams (g). During each weekly observation, a given mouse underwent 7 successive trials, with 5 to 10 seconds-intertrial intervals on the table. The mean T-PK of at least 5 successful trials, produced by all limbs was calculated and analyzed.

All behavioral apparatus were cleaned out with 70% ethanol after each test for each mouse. The observer was blind to genotype and treatment for all behavioral assessments.

#### Clasping Score

Clasping behavior was evaluated and scored as a semi-quantitative measure of limb dystonia [30]. Limb movements were videotaped when mice were suspended by the tail at a height of at least 30 cm for 20 seconds, followed by a brief touchdown and a subsequent suspension for another 20 seconds. Clasping was defined as a retraction of a limb towards the body and rated by an observer unaware of experimental groups. The extent of clasping at each limb was graded as follows: None = 0; mild = 0.25, when the fore- or hind- limb retracted toward the midline but did not reach the midline and the contraction was not sustained; moderate = 0.5, a high amplitude limb traction to or beyond the midline, but not sustained; severe and constant = 0.75, a high amplitude limb retraction sustained for more than 15 seconds. Fore- and hind- limbs clasping behavior was summed for each animal for a maximal clasping score of 3. The scores from the two tail suspension trials were then averaged and recorded.

#### Open field

Spontaneous locomotor activity of mice was examined weekly in an open field (50×50 cm square), from 4 weeks of age, using a video tracking system (Videotrack, Viewpoint Life Sciences, Montreal, Canada) with infrared backlighting [30], [35]. The system is able to analyse locomotor activity into different velocities as follows: slow movements (< 5 cm/sec); moderate movements (between 5-20 cm/sec); or fast movements (> 20 cm/sec). Total distance traveled by the animal during the 2 hours testing period, as well as the duration and distance covered at each velocity were measured. Locomotor activities were monitored continuously over the testing period, with output intervals of 3 minutes. A habituation session of 10 minutes preceded the locomotor activity monitoring when mouse was placed in the open field.

#### Elevated plus maze

The elevated-plus-maze was used to evaluate anxiety-like behaviors in experimental animals. The apparatus consisted of two open arms (each 44 cm×10 cm) and two closed arms of the same dimensions that extended from a common central platform (10 cm×10 cm). The maze was raised 90 cm above the floor under normal ambient overhead lighting conditions. Video-tracking system (ANY-MAZE, Stoelting) was used for the behavioral assessment in elevated plus maze. Mice at 8 weeks old were placed individually in the center square facing an open arm and allowed to explore the maze for 5 min. An arm entry was counted when all four paws were inside the arm. Exit from an arm was defined as when both forepaws left that arm. The time spent in the closed and open arms, the number of entries into the open and closed arms was tracked. Percent of time spent in open arm (time spent in the open arms as a proportion of time spent in all four arms) and percent of entries to open arm (entries to open arm as a proportion of total entries to all four arms) were used for analyses.

#### Barnes maze

Memory and learning were also evaluated using the Barnes maze [36], [37]. The maze consists of a circular platform (92 cm diameter) surrounded by 20 holes (5 cm diameter, equidistant), one providing an escape. The mouse was placed in a dark chamber in the center of the maze and aversive stimuli subsequently activated (bright light and noise). Prior to the first trial, each mouse was introduced to an adaptation period on day 1, in which it was conducted to the target hole allowing the escape box for 1 min. In the acquisition phase, mouse was subjected to 4 trials per day with an inter-trial interval of 20 min for four consecutive days. Each trial ended when the mouse entered the goal escape tunnel or for a maximum of 3 minutes. The reference memory phase or probe trials were conducted on the 5^th^ day and 12^th^ day following the first trials in order to test short- and long-term retention memory, respectively. During the probe trials, the escape route was removed and replaced with a standard hole through which the animal could not enter. The mouse was tested during a 90 seconds period. The time required to find the target hole was recorded as the primary latency using the ANY-MAZE video tracking system. Spatial cues with distinct shapes were placed around the maze and kept constant throughout the experiment.

#### Novel object recognition

Mice either at 4 or 9 weeks of age were used for the novel object recognition test. The test was carried out in the same experimental apparatus as described for open field. In order to investigate novel object recognition in mice at 9 weeks of life, an experimentally naïve batch of mice, including all experimental groups was introduced. During training, two identical objects were introduced to mice and they were allowed to explore them for 5 minutes. The objects were cleaned with ethanol 70% between trials to minimize olfactory cues. The first recognition test carried out one hour after the training. In this first trial which lasted for 5 minutes one of the objects were substituted for a novel one (new) while the other remained unchanged (familiar). The second trial was performed 24 hours after training in which the novel object was again replaced with a novel object of a different shape and the animal was allowed to explore them during 5 minutes. Movements were recorded by video-tracking system (ANY-MAZE, Stoelting). Results are expressed in percent of the time to visit the new object, defined as the time spent exploring the new object/total time exploring new plus familiar objects.

### Tissue Preparation for post-mortem analyses

Five weeks after rAAV2- injections (i.e. at the end of week 10), animals were deeply anaesthetized, perfused transcardially with RNAse free 0.9% heparinized saline followed by 4% paraformaldehyde in phosphate buffer and then decapitated. Brains were extracted, postfixed in 4% PFA for 48 hours, and passed sequentially through 15% and 30% phosphate buffered sucrose solutions. Brains were then cut into 40 µm coronal sections using a freezing microtome and free-floating sections were collected serially in 6 vials containing phosphate-buffered saline (0.1M PBS, pH 7.4).

### Immunofluorescence

In order to verify the site of injections, brain sections were submitted to GFP-DAPI double immunofluorescence staining and investigated under a fluorescent microscope. One set of brain sections from each animal were blocked in PBS containing 5% bovine serum albumin (BSA) and 0.3% Triton X-100 for 30 min. The sections were then incubated overnight at 4°C with the primary antibody, rabbit anti-GFP (1∶1000; Invitrogen) in PBS containing 2% BSA and 0.1% Triton X-100. Following 3 washes in PBS, fluorescent rabbit secondary antiserum (Alexa Fluor 488, 1∶500; Invitrogen) was added at room temperature for one hour, followed by DAPI (1:1000, Invitrogen) staining for 5 minutes. After last wash in PBS, the sections were mounted and then coverslipped with PermaFluor™ Aqueous Mounting Medium (Thermo Scientific).

### *In situ* hybridization

One set of brain sections from each animal were processed for *in situ* hybridizations under RNAase-free conditions. The sections were hybridized with [^35^S]UTP radiolabelled cRNA probes for detection of pENK mRNAs. The pENK probe (935 bp, from nucleotide −104 to 830) was generated from the linearized rat cDNA contained in pSP64 (antisense) and pSP65 (sense) plasmids [38]. The pENK cDNA template was linearized with *Eco*RI restriction enzyme, and the antisense probe was synthesized with [^35^S]UTP and T7 RNA polymerase [39].

Briefly, hybridization technique was performed as follows: after short fixation of 5 minutes in 4% PFA/PBS, free-floating sections were washed in PBS and incubated for 10 minutes in proteinase K/PBS, followed by second fixation in 4% PFA/PBS during 10 minutes. After washing with PBS and ddH2O, sections were incubated for 10 minutes in 0.25% acetic anhydride in 2% triethanolamine, and then washed in saline-sodium citrate (SSC) for 5 minutes. *In situ* hybridization was done at 58°C overnight in a standard hybridization buffer containing 50% formamide as previously described [30], [40]. Following stringency washes, sections were mounted onto Superfrost plus slides, air-dried and dehydrated during 2 minutes in 30%, 60%, and 100% ethanol. The slide-mounted tissue sections were then air-dried and exposed to radioactive sensitive films (Kodak, Biomax MR, New Haven, CT), together with ^14^C standard slides (American Radiolabeled Chemicals, Inc., St Louis), during 21 to 24 hours at room temperature. Films were scanned and analysis of striatum (divided in four subregions along a medial-lateral and dorso-ventral axis) was performed using public domain National Institutes of Health image analysis software (NIH ImageJ, Bethesda, MD), normalizing optical density using the corpus callosum as a reference for background activity.

### Immunohistochemistry

One set of coronal brain sections were submitted to NeuN immunostaining. Following incubation in NaBH_4_ (0.5% w/v) for 20 minutes, the sections were incubated in TBS solution containing 0.5% Triton X-100 and 0.03% H_2_O_2_ during 30 minutes. Sections were then blocked in solution containing 10% bovine serum albumin (BSA), 0.3% Triton X-100, and 5% normal goat serum (NGS) in TBS for 30 min at room temperature. Sections were then probed for 48 to 72 hours at 4°C with mouse NeuN monoclonal antibody (Invitrogen) diluted to 1:500 in TBS solution containing 2% BSA, 0.1% Triton X-100, and 2% NGS. They were then placed for 1 hour at room temperature in a solution containing biotinylated goat anti-mouse IgG (1:400, Jackson immune-research) and subsequently incubated in the TBS solution containing avidin–biotin peroxidase complex (Vectastain Elite ABC Kit; Vector Laboratories, Burlington, ON), for 1 hour at room temperature. All steps were followed by appropriate washes in TBS. Finally, the reaction was developed in 3,3′-diaminobenzidine tetrahydrochloride (DAB) solution (Sigma, St. Louis, MO) and 0.1% of 30% hydrogen peroxide (Sigma, St. Louis, MO) at room temperature. Following the DAB reaction, sections were mounted out of distilled water onto slides, air-dried, counterstained with 0.1% cresyl violet (Sigma, St. Louis, MO) (Nissl stain), dehydrated in ascending grades of ethanol, cleaned in xylene, and coverslipped with DPX (BDH Laboratories Supply), and kept until stereological quantification.

Enkephalin immunohistochemistry on free floating brain sections has also been used to estimate the intensity of enkephalin immunoreactivity in the striatum and the density of enkephalin containing striatal fibers [41], using rabbit leucine-enkephalin antibody (Immunostar, 1∶10000) and biotinylated donkey anti-rabbit IgG (1∶2000, Jackson immune-research). The reaction was developed in DAB solution and 0.1% of 30% hydrogen peroxide as described above. The sections were then mounted on slides, air-dried, and after dehydration in ascending grades of ethanol, they were cleaned in xylene, and coverslipped with DPX. Image analysis of enkephalin immunoreactivity at striatal levels, globus pallidus (GP) and substantia nigra (SN) was performed using public domain National Institutes of Health image analysis software (NIH ImageJ, Bethesda, MD). Optical densities were normalized to the respective white matter (e.g. corpus callosum, internal capsule as a reference for background activity) on each section.

### Stereology

Unbiased stereology was carried out on NeuN-Nissl stained coronal sections using the Stereo Investigator program (Microbrightfield, USA; V6) and a Nikon Eclipse 80i microscope equipped with a motorized XYZ stage. The optical fractionator was used to obtain unbiased estimates of the total number of neurons. The striatum was delineated according to defined boundaries [30], [42] using a stereotaxic atlas of the mouse brain [34]. Briefly, the selection includes levels throughout the striatum including regularly spaced sections caudal and rostral to the decussation of the anterior commissure. The dorsal, medial and lateral limits of the striatum are well defined [34]. Ventrally, the striatum interfaces with the amygdala and substantia innominata in its postcommissural part and with the nucleus accumbens in its precommissural division. The ventral limit of the striatum at the postcommissural part is well delineated on NeuN-Nissl stains. However, the ventral limit of the striatum at its precommissural part is an arbitrary interface. At the precommissural levels analyzed, we therefore delimit the dorsal striatum from the nucleus accumbens with a line that extends from above the ventral most part of the lateral ventricle medially, to the tapered external capsule laterally, at an angle of 25–30° below the axial plane. Section thickness was assessed and neuronal counts were performed under oil immersion using a 60× Plan Apo objective lens (1.40/0.17 DIC N2). The Cavalieri method was used to measure the striatal volume. The systemic random sampling grid size was 500×500 µm and the optical fractionator brick size was 80×80 µm. Neurons were defined as NeuN positive profiles measuring at least 5 µm in diameter. Glial cells are distinct as Nissl positive cells with diameter less than 7 µm, or Nissl positive cells with diameter more than 7 µm, light violet staining, and irregular shape as probable activated glia. The investigator was blind to the mouse treatment.

### Statistical analyses

Statistical analysis of data in behavioral and post-mortem experiments between WT and different groups of R6/2 mice were carried out by two-way or one-way analysis of variance (ANOVA) followed by *post hoc* analysis using Bonferroni and Dunn test (Stat-View, Abacus Corporation, Baltimore, MD, USA). Data are presented as a mean ± SEM. A *p*-value <0.01 was considered to be significant for all Bonferroni and Dunn *post hoc* analysis.

## Results

### The site of injections

**Figure 1:**
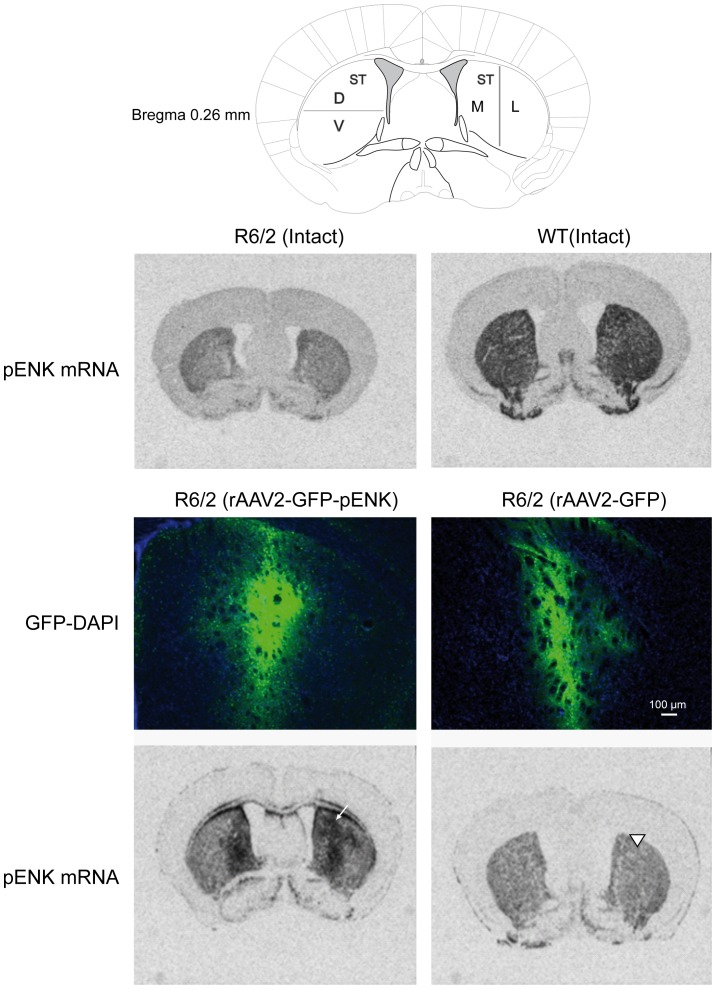
Representative images of the site of injection of rAAV2- in the striatum, revealed by GFP-DAPI double labeling, as well as representative autoradiograms of striatal pENK mRNA (*in situ* hybridization), compared to the schematic of the mouse brain (adapted from the atlas of Franklin and Paxinos, 2008). Arrow indicates the site of injection of rAAV2-GFP-pENK which corresponds to the overexpression of striatal pENK mRNA. Arrowhead indicates the site of injection of rAAV2-GFP without any changes in the expression of striatal pENK mRNA.

Representative images of the site of injection of rAAV2- in the striatum, revealed by GFP-DAPI double labeling, compared to the schematic of the mouse brain (adapted from the atlas of Franklin and Paxinos, 2008), are presented in Figure 1.

It should be noted that only mice with proper injection site were included in analysis.

### Striatal pENK mRNA expression

**Figure 2:**
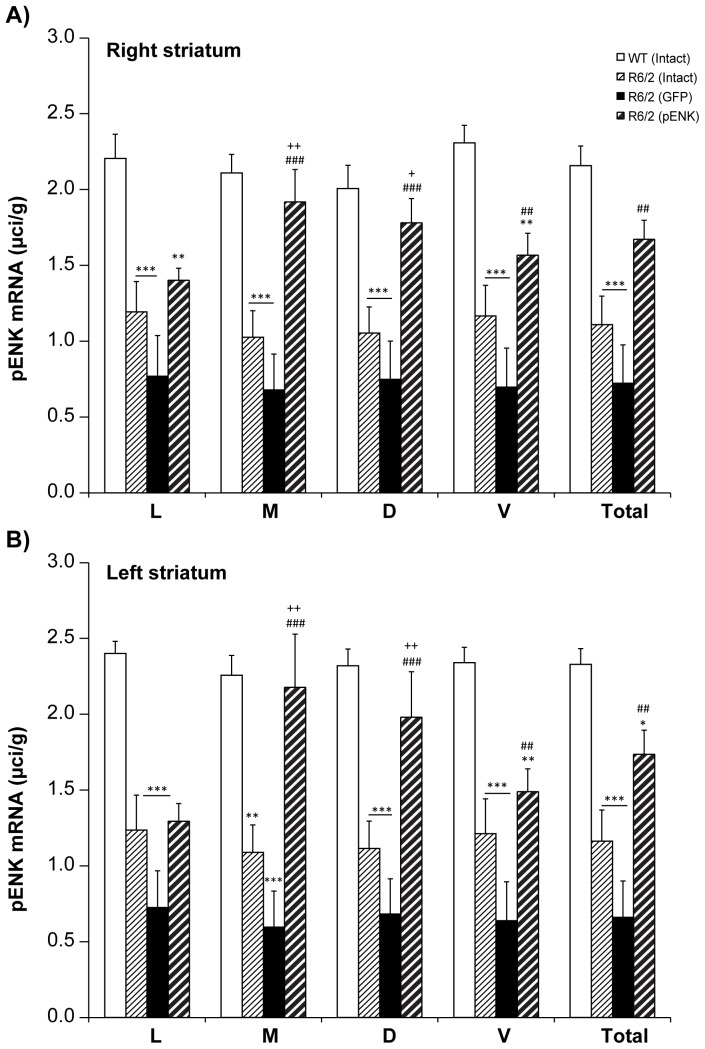
pENK mRNA expression in the striatum of different groups of R6/2 and WT mice. The level of pENK mRNA was increased in right (A) and left (B) striatum (total) of R6/2 mice having received striatal injection of rAAV2-pENK compared with intact and GFP-injected R6/2 mice. Moreover, upregulation of pENK mRNA was evident in all striatal subregions of pENK-injected mice compared with GFP-injected R6/2. The increased expression of pENK mRNA was more pronounced in medial and dorsal parts of the striatum. Indeed, in the medial and dorsal striatum no difference was observed between pENK-injected R6/2 mice and WT. L: lateral; M: medial; D: dorsal; V: ventral regions of the striatum. Data are presented as mean ± SEM. ** p<0.005, *** p<0.0005 vs. WT; ## p<0.005, ###p<0.0005 vs. R6/2 (GFP). + p<0.05, ++ p<0.005, +++ p<0.0005 vs. intact R6/2.

Representative autoradiograms of striatal pENK mRNA (*in situ* hybridization), compared to the schematic of the mouse brain are presented in Figure 1.Two-way ANOVA detected significant difference between different groups in the expression of striatal pENK mRNA, without any difference between right and left striatum [*F*(treatment)_3,31_ =  29.42, *p*<0.0001; *F*(right vs. left)_1,31_ = 0.11, *p* = 0.73, and *F*(treatment ×right vs. left)_3,31_ = 0.10, *p* = 0.96] [R6/2: intact (n = 5), GFP- (n = 4), pENK (n = 5); WT: (n = 5)] (Fig. 2A, B). *Post hoc* analysis indicated that pENK mRNA expression was significantly increased in the striatum of R6/2 mice having received rAAV2-pENK compared with GFP-injected and intact R6/2 mice (*p*<0.0001 and *p*<0.002, respectively). Further analysis in each hemisphere noted significant difference in different striatal subregions between different groups in the right [lateral: *p* = 0.0002; medial: *p* = 0.0002; dorsal: *p* = 0.0005; ventral: *p*<0.0001], and left [lateral: *p* = 0.0001; medial: *p* = 0.0007; dorsal: *p* = 0.0004; ventral: *p*<0.0001] hemispheres. Although pENK mRNA is increased in lateral striatum of pENK-injected R6/2 mice compared with GPF group but this difference did not reach significance (right and left: *p*>0.01). The level of pENK mRNA in lateral striatum of WT mice was higher than different groups of R6/2 mice at right (intact: *p* = 0.0004, GFP: *p*<0.0001, pENK: *p* = 0.002) and left (intact: *p* = 0.0001, GFP: *p*<0.0001, pENK: *p* = 0.0001) hemispheres (Fig. 2A, B). At medial and dorsal subregions of the striatum, concentration of pENK mRNA was higher in mice having striatal overexpression of pENK compared with intact and GFP-injected R6/2 mice at right [medial (intact: *p* = 0.0004, GFP: *p* = 0.002); dorsal (intact: *p* = 0.009, GFP: *p* = 0.001)] and left [medial (intact: *p* = 0.007, GFP: *p* = 0.0007); dorsal (intact: *p* = 0.01, GFP: *p* = 0.001)] hemispheres. Interestingly, no difference for the level of pENK mRNA was detected between WT and pENK-injected R6/2 mice at medial and dorsal striatum at right and left hemispheres. However, in WT mice pENK mRNA at medial and dorsal striatal subregions was higher than intact and GFP-injected R6/2 mice at right [medial (intact: *p* = 0.0009, GFP: *p* = 0.0001); dorsal (intact: *p* = 0.001, GFP: *p* = 0.0002)] and left [medial (intact: *p* = 0.004, GFP: *p* = 0.0004); dorsal (intact: *p* = 0.001, GFP: *p* = 0.0002)] hemispheres. No difference was observed between intact and GFP-injected R6/2 mice at medial and dorsal striatal subregions at right and left hemispheres (Fig. 2A, B). pENK mRNA is also increased in ventral striatal region of pENK-injected R6/2 mice compared with GPF- injected mice (right: *p* = 0.005; left: *p* = 0.006), but this increase did not reach significance compared with intact R6/2 group. In WT mice the concentration of pENK mRNA in ventral part of the striatum was higher than different R6/2 groups at right (intact: *p* = 0.0002, GFP: *p*<0.0001, pENK: *p* = 0.005) and left (intact: *p* = 0.0004, GFP: *p*<0.0001, pENK: *p* = 0.002) hemispheres (Fig. 2A, B).

### Behavioral characterization

Behavioral assessments began at week 4, one week prior to the striatal delivery of rAAV2- (on week 5) to ensure that the behavioral alterations possibly observed in following weeks were not due to pre-existing differences among groups. In order to avoid the confounding effects of a given test on the performance of mice in another, the timing of the tests were carefully selected. Specially, Barnes maze and Novel object preference tests were carried out at least one week apart.

### Weight gain

**Figure 3:**
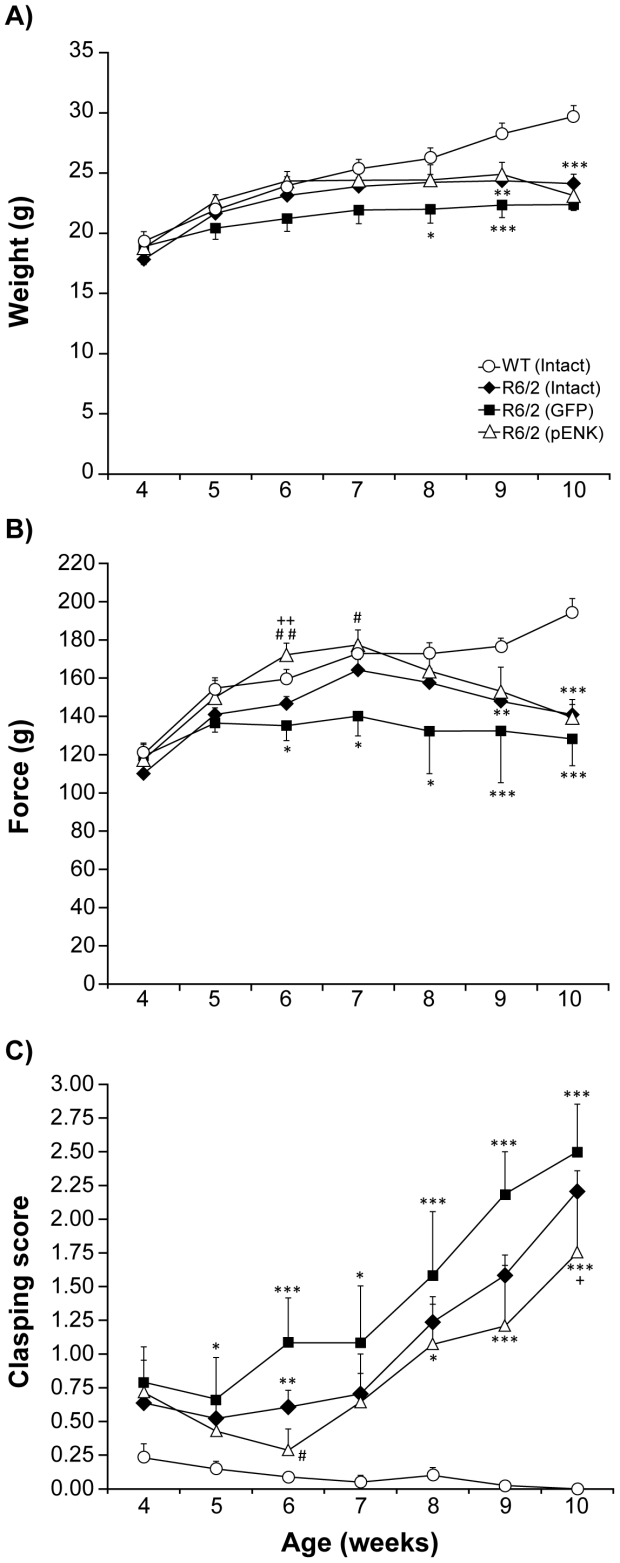
Weight, muscular force, and abnormal clasping movement of different groups of R6/2 mice and WT at different ages. At week 10, all groups of R6/2 mice displayed significantly lower weights compared to their WT counterparts. However, the failure in weight gain started sooner in R6/2 mice having received striatal rAAV2-GFP compared with pENK-injected mice. Interestingly, the decline in weight gain was delayed until week 10 in pENK- R6/2 mice compared to WT (A). The muscle force was recorded for all for- and hind- limbs as tension-peak (T-PK) to grasp grid mesh, using Grip Strength. GFP-injected R6/2 mice had an overall worse performance than other R6/2 mice. However, striatal overexpression of pENK improved the muscular force of R6/2 mice and delayed considerably the onset of decline in all limbs force (B). Higher clasping score was observed in GFP-injected R6/2 mice at different ages. However, striatal overexpression of pENK delayed the onset of this abnormal movement until week 8 compared with WT. Moreover, clasping score in pENK-injected R6/2 mice was less than GFP- injected group at 6-old-week (C). Data are presented as mean ± SEM. ** p<0.01, and *** p<0.0001 vs. WT; + p<0.01 vs. R6/2 (Intact); # p<0.01, ## p<0.01, ### p<0.005 vs. R6/2 (GFP).

The animals were weighed once weekly and the average weight in each week was compared among groups. Significant differences were noted when different groups of mice were compared at different ages [*F*(groups)_3,241_ = 17.84, *p*<0.0001; *F*(age)_6,241_ = 17.37, *p*<0.0001, and *F*(groups×age)_18,241_ = 2.01, *p* = 0.009] [R6/2: intact (n = 11), GFP- (n = 4), pENK (n = 6); WT: (n = 17)]. *Post hoc* analysis showed a significant difference between: WT mice compared to the intact (*p*<0.0001), GFP- (*p*<0.0001), and pENK- (*p* = 0.0002) injected R6/2 groups; as well as pENK- R6/2 mice compared to GFP- injected group (*p* = 0.01). Further analysis demonstrated that the weight in all experimental groups was similar at 4 weeks of age (*p* = 0.64), however WT mice gained more weight over time compared to R6/2 groups (Fig. 3A). Moreover, striatal injection of rAAV2-GFP further accelerated the disturbance in weight gain of R6/2 mice over time, such that in these mice the failure in weight gain started from 8 weeks of age (*p* = 0.01 vs. WT), while weight gain was impaired in intact R6/2 mice from week 9 (*p* = 0.001 vs. WT). Interestingly, the decline in weight gain was delayed until week 10 in pENK- R6/2 mice compared to WT (*p* = 0.0002) (Fig. 3A).

### Grip strength

The muscle force exerted by all limbs to grasp grid mesh was measured using Grip Strength. Tension peak (T-PK) forces were recorded and compared as a criterion for animal muscular strength. T-PK generated when mice held the wire grid mesh using all limbs was significantly different between groups at different ages [*F*(groups)_3,241_ = 23.08, *P*<0.0001; *F*(age)_6,241_ = 12.95, *P*<0.0001, and *F*(groups×age)_18,241_ = 2.54, *p* = 0.0007] [R6/2: intact (n = 13), GFP- (n = 4), pENK (n = 6); WT: (n = 17)]. *Post hoc* analysis noted greater muscular force in WT mice compared to intact (*p*<0.0001), GFP- (*p*<0.0001), and pENK- (*p* = 0.005) injected R6/2 mice. It is interesting to note that impaired grip strength was even more pronounced in GFP group than intact (*p* = 0.01), and pENK- (*p* = 0.0001) injected R6/2 groups. As illustrated in Figure 2B, one-way ANOVA followed by Bonferroni *post hoc* test detected a progressive impairment in muscular force of all limbs in all studied ages from week 6 to 10 in GFP-injected R6/2 group compared to WT (*p* = 0.01; *p*<0.0001, respectively). Although GFP-injected R6/2 mice had an overall worse performance than intact R6/2 mice, but this difference did not reach significance at any ages. Compared with WT mice, the decline in all limbs force in intact R6/2 mice emerged at weeks 9 and 10 (*p* = 0.005 and *p*<0.0001, respectively) (Fig. 3B). Interestingly, striatal overexpression of pENK had a beneficial impact on the muscular force of R6/2 mice by delaying the onset of decline in all limbs force. Indeed, the difference between pENK-injected R6/2 mice and WT was reached significant level only at week 10 (*p* = 0.0001). Moreover, the improvement of T-PK produced by all limbs in pENK-injected R6/2 mice was significant at weeks 6 and 7 compared with GFP-injected group (*p* = 0.002, *p* = 0.01, respectively), and at week 6 compared with intact R6/2 mice (*p* = 0.005) (Fig. 3B).

### Clasping phenotype

Clasping was scored as a dystonic movement illustrated by the abnormal retraction of limbs toward the body. Two way ANOVA showed a difference for involuntary clasping scores between different groups of mice at different ages [*F*(groups)_3,204_ = 66.52, *P*<0.0001; *F*(age)_6,204_ = 12.61, *p*<0.0001, and *F*(groups×age)_18,204_ = 3.63, *p*<0.0001] [R6/2: intact (n = 8), GFP- (n = 4), pENK (n = 6); WT: (n = 15)]. *Post hoc* analysis noted a difference between WT and each of R6/2 groups (*p*<0.0001). In addition, clasping score was different between pENK-injected R6/2 mice compared with intact (*p* = 0.01), and GFP- (*p* = 0.0002) mice. Further analysis using one way ANOVA followed by Bonferroni *post hoc* test showed that clasping in intact and GFP-injected R6/2 mice was higher than WT littermates from week 5 (*p* = 0.01) and then progressively increased with age until week 10 (*p*<0.0001) (Fig. 3C). Interestingly, the highest expression of abnormal clasping movement was observed in GFP-injected R6/2 mice at different ages (Fig. 3C). However, overexpression of pENK in the striatum delayed the onset of this abnormal movement until week 8 compared with WT (*p* = 0.01). Moreover, the improvement of clasping movement in pENK-injected R6/2 mice was evident at weeks 6 compared with GFP-injected group (*p* = 0.01), and at 10 weeks old compared with intact R6/2 mice (*p* = 0.01) (Fig. 3C).

### Locomotor activity in open field

#### Total distance travelled

**Figure 4:**
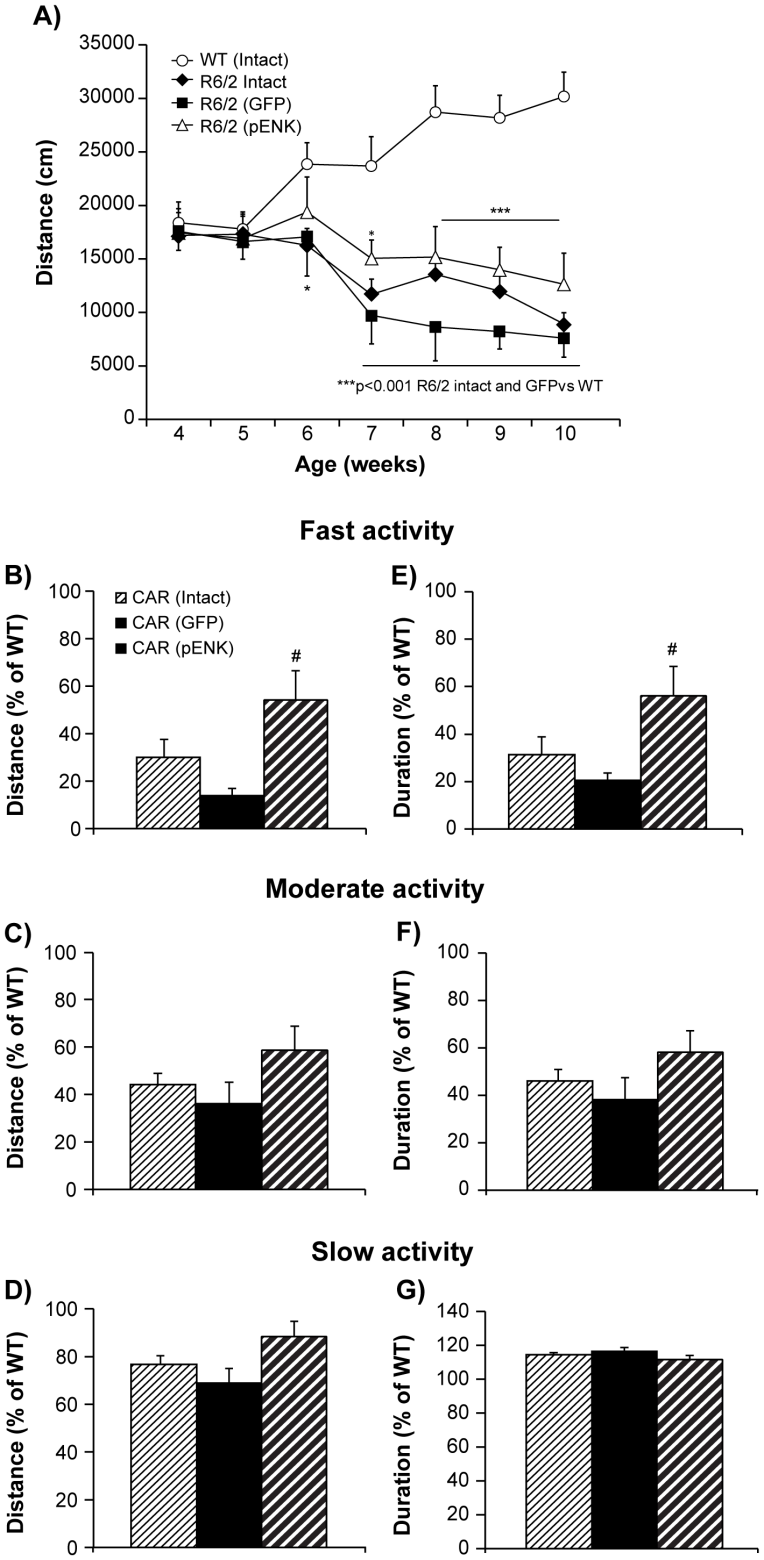
Spontaneous locomotor activity, distance travelled, and duration spent at various movement velocities in different groups of R6/2 mice compared with WT. The total distance traveled by mice in open field, during 2/2 mice compared with WT (A). However, the reduction of locomotor activity was more pronounced in GFP-injected group. The distance (B) and duration of time (E) spent in fast speed was higher in pENK-injected compared with GFP-injected R6/2 mice. While the distance and duration of time spent in moderate speed (C, F) and slow speed (D, G) tended to be higher in pENK-injected R6/2 compared with other R6/2 groups, however this difference did not reach significance. Data are presented as mean ± SEM. * p<0.01, *** p<0.001 vs. WT; # p<0.01 vs. R6/2 (GFP).

When the total distance traveled during 2 hours was analyzed in terms of groups and age, a significant difference was observed between different groups of mice as well as their interaction [*F*(groups)_3,258_ = 49.73, *p*<0.0001; *F*(age)_6,258_ = 1.33, *p* = 0.24, and *F*(groups×age)_18,258_ = 3.95, *p*<0.0001] [R6/2: intact (n = 14 to 10), GFP- (n = 5 to 4), pENK (n = 6); WT: (n = 17)]. *Post hoc* analysis noted a difference between WT and each of R6/2 groups (*p*<0.0001). Further analysis showed that the total distance travelled during two hours was not different between different groups at weeks 4 and 5 (F_3,38_ = 0.09, p = 0.97; F_3,38_ = 0.07, p = 0.98, respectively) (Fig. 4A). However, a significant difference was observed between R6/2 mice and aged-matched WT mice from weeks 6 to 10 (F_3,38_ = 2.92, p = 0.04; F_3,31_ = 22.72, p<0.0001, respectively). The reduction of distance travelled by intact R6/2 mice emerged at week 6 compared to WT control (*P* = 0.007) and was sustained in all ages. The total distance travelled during two hours was also reduced from weeks 7 to 10 in R6/2 mice receiving either rAAV2-GFP (*p* = 0.0002, *p*<0.0001) or rAAV2-GFP-pENK (*p* = 0.008, *p*<0.0001) compared to age-matched WT mice. There was no difference among the R6/2 mice in various groups (Fig. 4A).

#### Distance travelled and time elapsed at various movement velocities

As movement with various velocities is differently affected in R6/2 mouse model of HD [30], different movement speeds were compared between different groups of mice.

A comparison of total distance traveled during fast activity from weeks 6 to 10 (as % of aged-matched WT) revealed a significant difference between different groups of R6/2 mice (*F*_2,22_ = 4.73, *p* = 0.01) (Fig. 4B). Indeed, *post hoc* analysis showed an improvement of fast activity in pENK-injected R6/2 mice (54% of WT) compared with GFP-injected group (14% of WT) (*p* = 0.007). While the distance traveled during moderate (Fig. 4C) and slow (Fig. 4D) activity was higher in pENK-injected (59% and 88% of WT) compared with GFP- (36%, 69% of WT) and intact (44%, 77% of WT) R6/2 mice, however this difference did not reach significance (*F*_2,22_ = 1.70, *p* = 0.21 and *F*_2,22_ = 2.00, *p* = 0.16, respectively).

The duration of time spent in fast speed (percent of aged-matched WT group) was significantly different between various groups of R6/2 mice (*F*_2,22_ = 3.88, *p* = 0.03). From weeks 6 to 10, R6/2 mice having received striatal rAAV2-GFP-pENK spent significantly longer time in fast activity (56% of WT) compared with rAAV2-GFP (21% of WT) R6/2 mice (*p* = 0.01) (Fig. 4E). While pENK-injected R6/2 mice (58% of WT) spent more time in moderate velocity than GFP- (38% of WT) and intact (46% of WT) groups (Fig. 4F), however this difference did not reach significance (*F*_2,22_ = 1.21, *p* = 0.32). No difference in time moving at slow velocity, from weeks 6 to 10, was observed between pENK-injected (112% of WT), GFP-injected (117% of WT), and intact (115% of WT) R6/2 mice (*F*_2,22_ = 1.09, p = 0.35) (Fig. 4G).

### Elevated plus maze

**Figure 5:**
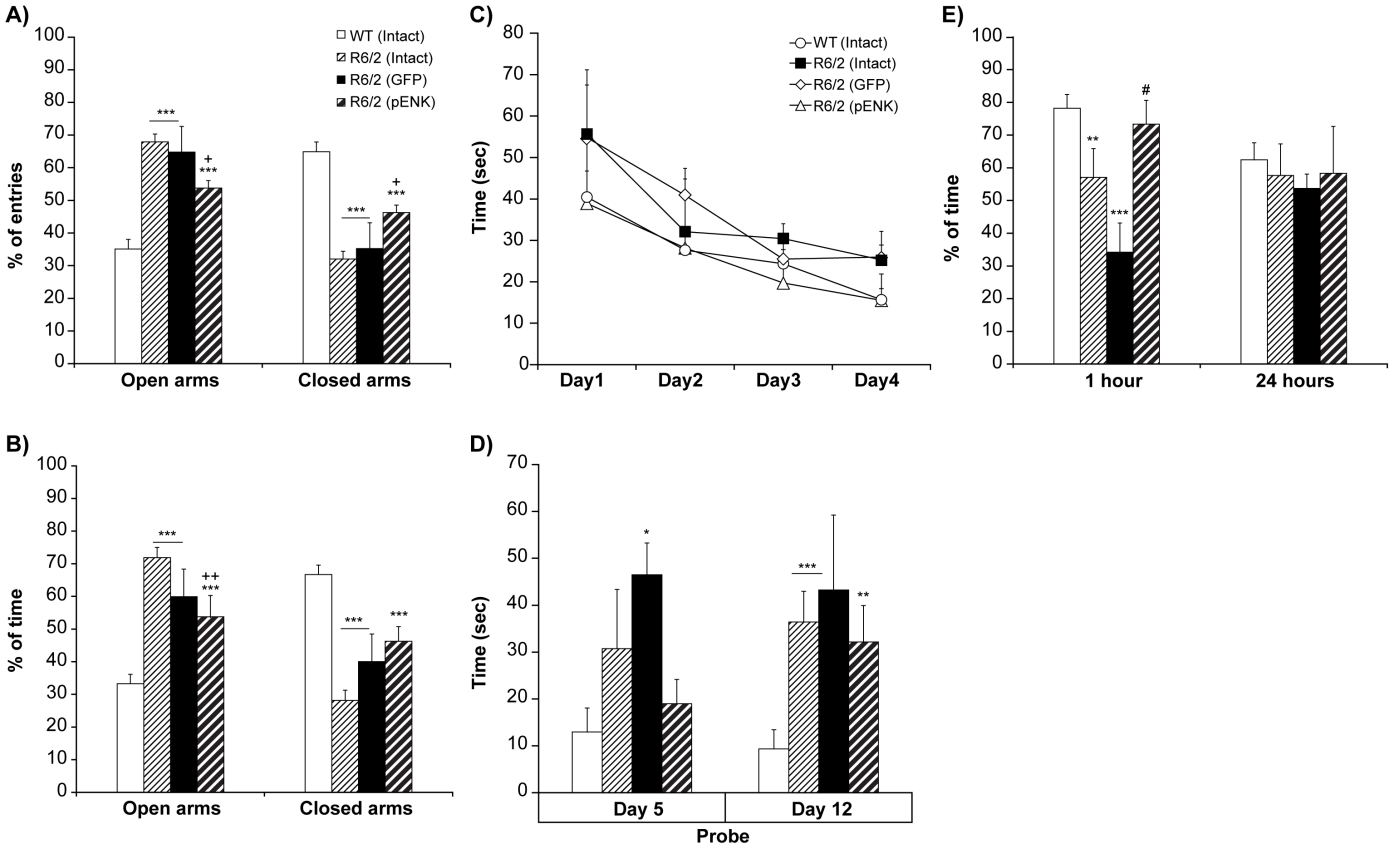
Elevated plus maze, Barnes maze, and Novel object recognition test in different groups of R6/2 and WT mice. Entry and time spent in open and closed arms of elevated plus maze (A, B): At week 8 of age, all R6/2 mice showed more percentage of entries (A), and spent more time in the open arm (B) compared with WT. In R6/2 mice having striatal overexpression of pENK, a reduction in percent of entries (A) and percent of time elapsed (B) in the open arms was observed compared with intact age-matched R6/2 mice. The percentage of entries and the proportion of time spent in the closed arms was exactly the reverse of what was seen for the open arms. Data are presented as mean percent of entries or mean percent of time spent in the open or closed arms / time spent in all four arms ± SEM. *** p<0.0005 vs. WT; + p<0.01, ++ p<0.005 vs. R6/2 (Intact). Learning and memory in Barnes maze (C, D) and Novel object recognition test (E): In Barnes maze performance, started at week 7 of age, no difference was observed in latency to find escape hole between different groups during trial days (C). At probe day 5^th^ (D), R6/2 mice having striatal injection of rAAV2-GFP showed increased latency to find target hole compared to WT and pENK-injected R6/2 mice. Whereas no difference was detected between pENK-injected R6/2 mice and WT, while intact R6/2 mice tended to spent longer time to reach the target hole compared with WT counterparts. At probe day 12^th^ (D), the time latency to find target hole was higher in all groups of R6/2 than WT. Novel object recognition test, at week 9, showed that in 1 hour retention test (E), intact R6/2 mice spent less time to explore the novel object compared to WT. Less preference toward novel object was even more pronounced in GFP-injected R6/2 mice compared with WT and pENK-injected group. Interestingly, recognition of novel object was considerably improved in R6/2 mice having striatal overexpression of pENK. Data are presented as percent of the time spent exploring the new object/ total time exploring new and familiar objects ± SEM. * p<0.01, ** p<0.005, *** p<0.0005 vs. WT; + p<0.01, ++ p<0.005 vs. R6/2 (Intact); # p<0.01 vs. R6/2 (GFP).

The elevated plus maze was employed to assess the anxiety in different groups of R6/2 mice (Intact: n = 14; GFP: n = 4; pENK: n = 6) compared with WT (n = 17) at 8 weeks old (3 weeks after striatal rAAV2- injections). Statistical analysis detected a significant difference between various groups for percentage of entries (*F*_3,37_ = 37.55, *p*<0.0001) (Fig. 5A) and proportion of time (*F*_3,37_ = 38.36, *p*<0.0001) (Fig. 5B) spent in the open arm. Interestingly, irrespective of their treatments, all R6/2 mice (intact, GFP- and pENK-injected) made significantly more percentage of entries onto the open arm, and spent more proportion of time in the open arm compared with their WT littermates (intact and GFP groups *P*<0.0001, pENK *p* = 0.0002) (Figs. 5A,B), suggestive of a lower level of anxiety in R6/2 genotype. Of importance, striatal pENK overexpression induced a noticeable reduction in proportions of entries and elapsed time in the open arms compared with intact age-matched R6/2 mice (respectively *p* = 0.008 and *p* = 0.002) (Figs. 5A,B). In addition, the percentage of entries and the proportion of time spent in the closed arms was exactly the reverse of what was seen for the open arms (Figs. 5A,B).

### Barnes maze

The Barnes maze test was initiated at week 7 to examine the striatum-dependent memory and learning. Following four days of training, two probe trials were performed on days 5^th^ and 12^th^ to test short- and long-term retention memory. During the course of the training (days 1 to 4), all mice learned to find the target hole as indicated by a progressive reduction in primary latencies. No difference was detected in the latency times to find the target hole over the training period among the experimental groups (*F*_3,27_ = 1.47, *p* = 0.25) [R6/2: intact (n = 7), GFP- (n = 4), pENK (n = 6); WT: (n = 14)] (Fig. 5C). However, a significant difference was observed between different groups for latency to find target hole on days 5^th^ and 12^th^ [*F*(groups)_3,54_ = 12.39, *p*<0.0001; *F*(probe)_1,53_ = 0.46, *p* = 0.50, and *F*(groups×probe)_3,54_ = 0.91, *p* = 0.44]. Further analysis on probe day 5^th^ noted a significant difference in primary latency among the groups (F_3,27_ = 4.53, *p* = 0.01) (Fig. 5D). Indeed, intact R6/2 mice spent longer time to reach the target hole than their WT counterparts but this difference did not reach significance (*p*>0.01). Of great note, striatal injection of rAAV2-GFP further worsened the maze performance in probe day 5^th^ compared to WT (*p* = 0.002). Interestingly, striatal pENK overexpression corrected the short memory disturbance seen in R6/2 mice (*p* = 0.48 vs. WT) (Fig. 5D). In probe day 12^th^, there was a significant difference in primary latency to find the target hole among groups (*F*_3,27_ = 9.85, *p* = 0.0001). At day 12^th^, intact and GFP-injected R6/2 mice showed disturbance in long-term memory compared with WT (*p* = 0.0003). Unlike its action in short-term memory, nonetheless, striatal pENK overexpression failed to provide a beneficial effect in long-term retention test (*p* = 0.002 vs. WT) (Fig. 5D).

### Novel object recognition task

This cognitive test was performed to assess the memory function by means of preference for a novel object in different experimental groups. Different groups were tested at week 4 (before manipulation) and week 9 (4 weeks after rAAV2- injections in the striatum). At 4 weeks of age, R6/2 showed similar exploratory behavior to WT in training session as well as in 1 hour and 24 hours retention trials (data not shown). Moreover, at 9 weeks of age, during the training session, no preference was seen for two identical objects among different experimental groups (*F*_3,19_ = 0.82, *p* = 0.50). However, a significant difference was detected between different groups of mice for exploring a new object in retention trials at 1 or 24 hours [*F*(groups)_3,38_ = 5.95, *p* = 0.002; *F*(hours)_1,38_ = 0.69, *P* = 0.41, and *F*(groups×hours)_3,38_ = 1.19, *p* = 0.33]. Further analysis detected a significant difference between various groups of [R6/2: intact (n = 6), GFP- (n = 3), pENK (n = 3); WT: (n = 12)] in 1 hour retention test (*F*_3,19_ = 5.24, *p* = 0.003). At this age (week 9), intact R6/2 mice spent a significantly less time exploring the novel object compared to WT (*P* = 0.008). GFP-injected R6/2 mice also displayed a recognition deficit compared with WT and pENK-injected animals (*p* = 0.001 and *p* = 0.01, respectively) (Fig. 5E). In addition, GFP- R6/2 mice tended to show less preference toward novel object than the intact R6/2 but this difference did not reach the significance level. In sharp contrast, novel object recognition was significantly improved in R6/2 mice receiving striatal rAAV2-GFP-pENK injection compared to GFP-injected R6/2 mice. It appears that striatal pENK overexpression efficiently rescues recognition function in R6/2 mice as there was no difference in novel object discrimination between the latter and their WT littermates (*p* = 0.49) (Fig. 5E). The novel object exploration time did not vary among experimental groups at 24 hours retention test (F_3,19_ = 1.40, *p* = 0.27) (Fig. 5E).

### Enkephalin intensity in the striatum and striatal fibers

**Figure 6:**
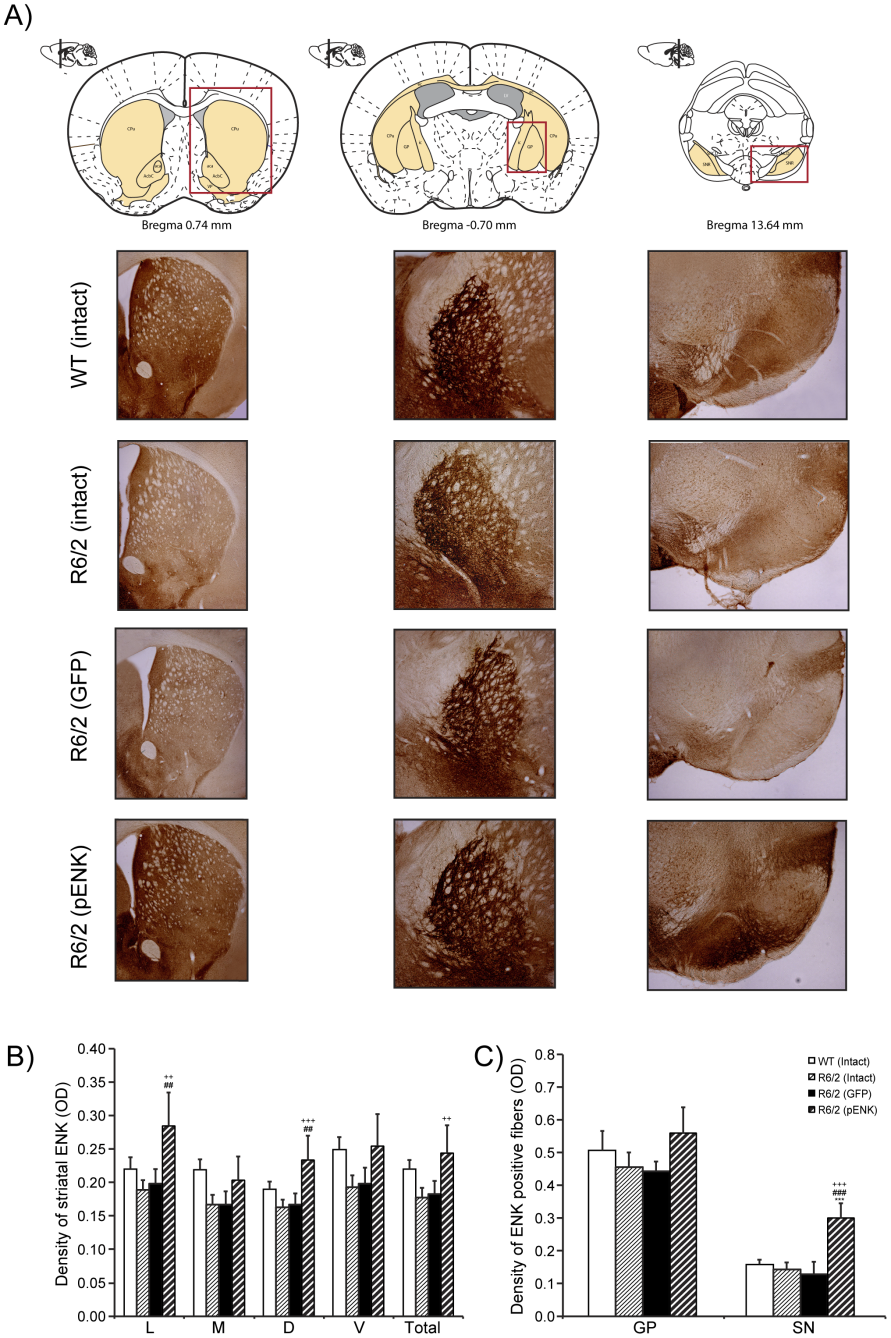
Enkephalin immunoreactivity in the striatum, globus pallidus and substantia nigra of different groups of R6/2 and WT. Representative images of immunohistochemistry against enkephalin in the striatum, GP and SN in different groups (A). The density of enkephalin positive neurons was increased in the total striatum of pENK-injected R6/2 mice compared with intact R6/2 mice. Moreover, the increased density of enkephalin in the lateral and dorsal subregions of the striatum was higher in pENK-treated group compared with intact and GFP-injected R6/2 mice (B). No significant difference was observed for the density of enkephalin positive fibers in the GP between different groups; however, the density of enkephalin positive fibers was importantly increased in the SN of R6/2 mice having striatal overexpression of pENK (C). L: lateral; M: medial; D: dorsal; V: ventral regions of the striatum; GP: globus pallidus; SN: substantia nigra. Data are presented as mean ± SEM. **** p<0.0001 vs. WT; ## p<0.005, ####p<0.0001 vs. R6/2 (GFP). ++ p<0.005, +++ p<0.0005, ++++ p<0.0001 vs. intact R6/2.

Representative images of immunohistochemistry against enkephalin in the striatum, GP and SN in different groups of R6/2 and WT mice [R6/2: intact (n = 8), GFP- (n = 4), pENK (n = 5); WT: (n = 7)] are presented in Figure 6A. Two-way ANOVA detected significant difference in enkephalin density at dorsal, lateral and total striatum between different groups [dorsal: *F*(treatment)_3,40_ =  5.27, *P*<0.004; lateral: *F*(treatment)_3,40_ =  5.00, *p*<0.004: total: *F*(treatment)_3,40_ =  3.85, *p*<0.01], while the difference between right and left striatum and the interaction for treatment ×right vs. left hemisphere was not significant (Fig. 6B). *Post hoc* analysis indicated that enkephalin intensity was significantly increased in the dorsal and lateral striatum of R6/2 mice having received rAAV2-pENK compared with GFP-injected and intact R6/2 mice (dorsal: *p* = 0.0005 and *p* = 0.004; lateral: *p* = 0.0006 and *p* = 0.006, respectively). Moreover, the level of enkephalin density was higher in total striatum compared with R6/2 (intact) (*p* = 0.004) (Fig. 6B). Although the density of enkephalin positive fibers in GP of pENK-injected R6/2 mice was higher than other groups of mice, but this difference did not reach significance (Fig. 6C). As the GP receives ENK-containing neurons from the striatum we suggest that the immunoreactivity chromogenbased has already reached saturation level in mice having striatal overexpression of pENK, and that's why despite the higher level of ENK in GP of pENK-injected animals this difference did not reach significance. Interestingly, the density of enkephalin positive fibers was different in the SN of different treated groups [*F*(treatment)_3,40_ =  14.24, *p*<0.0001; *F*(right vs. left)_1,40_ = 0.44, *p* = 0.51, and *F*(treatment ×right vs. left)_3,40_ = 0.37, *p* = 0.77]. Importantly, *Post hoc* analysis detected higher density of enkephalin positive fibers in the SN of R6/2 mice having striatal overexpression of pENK, not only compared with R6/2 intact and GFP-injected groups (*p*<0.0001, +52.2%, +57.2%, respectively), but also compared with WT (*p*<0.0001,) +47.2%) (Fig. 6C).

### Stereological analyses

**Figure 7:**
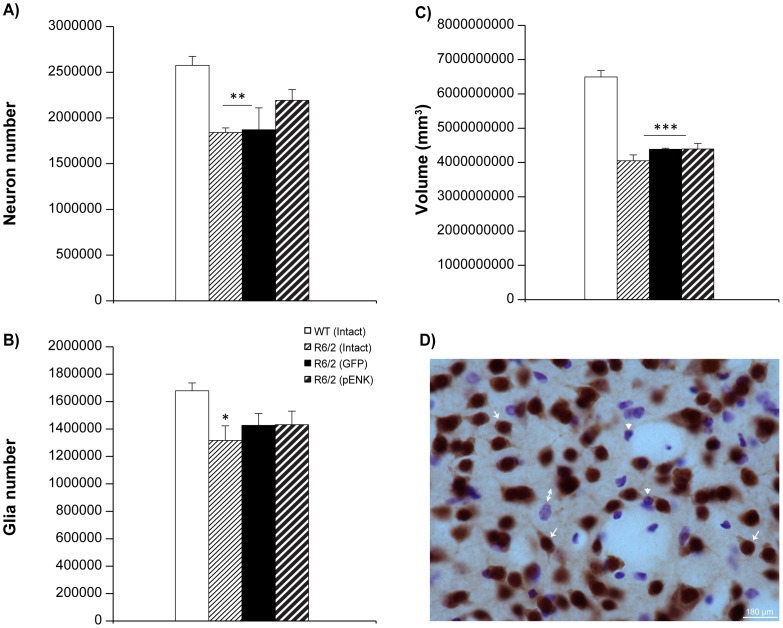
Morphological changes in the striatum of different groups of R6/2 mice and WT at week 10 of age. Unbiased stereology revealed that the total number of striatal NeuN positive neurons (A) was reduced in the striatum of intact and GFP-injected R6/2 mice compared with WT. However, this difference did not reach significance when WT mice were compared with pENK-injected R6/2 mice. The number of glial cells was higher in WT compared with intact R6/2 mice (B). The volume of the striatum was reduced in all groups of R6/2 mice compared with WT (C). Representative coronal brain sections stained with NeuN-Nissl for unbiased stereology (D); arrow: NeuN positive (neuron), arrowhead: Nissl positive (glia, <7 µm), and two headed arrows: Nissl positive (glia, >7 µm). Data are presented as mean ± SEM. * p<0.01, ** p<0.001, *** p<0.0001 vs. WT.

Unbiased stereological counts using optical fractionator method has been used to investigate the changes in the number of striatal neurons and glia, as well as striatal volume of different groups of R6/2 mice compared with WT. Indeed, in agreement with other studies [30], [31], our results confirmed the presence of striatal neuronal loss in R6/2 transgenic mouse model of HD, revealed by a difference for the number of NeuN positive neurons between various experimental groups (*F*_3,17_ = 7.30, *p* = 0.002) [R6/2: intact (n = 5), GFP- (n = 4), pENK (n = 6); WT: (n = 6)]. *Post hoc* analysis showed a higher number of NeuN positive neurons in the striatum of 10 weeks old WT mice compared with those from intact (*p* = 0.0007, 71% of WT), and GFP-injected (*p* = 0.001, 72% of WT) R6/2 mice. Nevertheless, this difference did not reach significance when WT mice were compared with pENK-injected R6/2 mice (*p*>0.01, 85% of WT) (Fig. 7A). In addition, at 10 weeks old, a difference was noted in the number of glial cells between different groups (*F*_3,17_ = 3.12, *p* >0.01) when all Nissl positive cells were taking into account. *Post hoc* analysis indicated that the number of glial cells was higher in WT compared with intact R6/2 mice (*P* = 0.01, 78% of WT). However, no change was observed in the number of Nissl positive cells between WT and GFP- or pENK-injected R6/2 mice (*p*>0.01) (Fig. 7B). Moreover, the volume of the striatum was significantly different between various groups of mice at 10 weeks old (*F*_3,17_ = 50.48, *p*<0.0001). In fact, in all groups of R6/2 mice the volume of the striatum was smaller than age-matched WT (*p*<0.0001) (Fig. 7C).

## Discussion

In the present study, we investigated whether striatal overexpression of pENK can reduce or delay behavioral dysfunction and/or striatal neuronal loss in R6/2 transgenic mouse model of HD. Our results, to our knowledge are the first to show that increased enkephalin transmission in the striatum could delay the progression of behavioral symptoms of HD in R6/2 mouse model by: a) improvement and delaying the onset of decline in grip strength which is one of the behavioral dysfunction in R6/2 mouse model of HD due to progressive loss in muscular force [30], [31]; b) reduction of clasping as an abnormal dystonic movement which is present in this mouse model and its severity increases with age [30], [31]; c) improvement of fast motor activity which is reduced considerably, more than moderate and slow activities, in R6/2 mice [30]; d) reduction of the percentages of entries and time spent in the open arms which is usually more elevated in R6/2 mice compared with WT [43]; e) improvement of short-term memory and recognition function which is altered in all mouse models of HD [29], [44], as well as in HD patients even before the onset of motor symptoms [45]. It is also important to note that the surgery by itself (e.g. decreased of food consumption after surgery) might be the cause of worsening HD-like symptoms in GFP-injected R6/2 mice, even compared to R6/2 intact. Interestingly, striatal pENK overexpression could overcome and delay the effect of surgery in weight loss, as well as decline in different behavioral functions compared to GFP-injected mice. At 10 weeks old, pENK striatal overexpression had also slight beneficial effect on striatal neuronal number compared with other groups of R6/2.

### Striatal pENK overexpression, density of enkephalin in the striatum, and behavioral symptoms in R6/2 mice

An important question that could be raised here is how pENK overexpression in the striatum could have beneficial effects on HD symptoms in the R6/2 mouse model of HD? One of the first neurodegenerative changes in HD is the loss of striatal medium spiny neurons with the preferential and earlier degeneration of striatopallidal neurons containing opioid peptide enkephalin [26], [46]. This concept is reinforced by data suggesting that striatal neurons containing enkephalin receive more glutamatergic cortical inputs and are more directly affected by cortical activity [46], [47], despite the more abundant expression of huntingtin in striatonigral projection neurons [48]. The effect of excitatory glutamatergic inputs to the striatum can be regulated by presynaptic receptors on corticostriatal terminals [46]. Nevertheless in HD, cellular pathology induced by mutant huntingtin in the presynaptic terminals might result in an increased release of glutamate [46], [49]. Indeed, the primary proposed cellular mechanism underlying degeneration of medium spiny neurons is the overactivity of corticostriatal glutamate transmission [46], [49]–[51]. Therefore, plausible approaches to reduce the glutamate release from corticostriatal terminals might be relevant in the treatment of HD. Accordingly, surgical lesions of the corticostriatal pathways, administration of glutamate release inhibitors or glutamate transporter upregulators, increased striatal neuronal survival and improved behavioral and biochemical phenotypes in R6/2 mice [52]–[55]. Interestingly, it was shown that the main action of enkephalin on striatal neurons, mainly through δ opioid receptors (δORs), is to provide the presynaptic inhibition of corticostriatal excitatory synaptic input [56], [57]. Therefore, we suggest that reduction of corticostriatal signalling *via* activation of presynaptic δORs through enkephalin released from striatal collaterals [58], might play an important role at delaying or attenuating motor dysfunction (e.g. locomotor activity, abnormal clasping, muscular force) in R6/2 mice having striatal overexpression of pENK. Indeed, this suggestion corroborates with our results concerning the increased density of striatal enkephalin in pENK-injected R6/2 mice.

Striatum has also a crucial role not only in the control of movement but also in instrumental learning [59], working memory and reversal learning [60]. In HD, cognitive deficits are even detectable prior to motor dysfunction, first in memory functions and then in executive functions [29], [44]. Interestingly, deficits in recognition memory and spatial memory induced by mutant huntingtin may be caused, at least in part, by protein kinase-A (PKA) over-activation [61]. It was shown that cAMP signaling cascade is increased in the striatum of R6 mice [62], and PKA substrates are hyper-phosphorylated in the striatum of mouse models of HD at pre-symptomatic stages [63]. Since one of the actions of enkephalin at the cellular level is inhibition of adenylate cyclase and reduction of intracellular cAMP level and signaling cascade [9], [64], therefore, it is possible to speculate that striatal pENK overexpression *via* activation of opioid receptors and reduction of cAMP level and/or PKA activity could play a role in the improvement of cognitive symptoms (e.g. short-term memory and recognition) observed in pENK-injected R6/2 mice.

Imbalance in Ca^++^ homeostasis and regulation have been implicated in the pathogenesis of many neurodegenerative diseases including HD. Importantly, striatal neurons are particularly vulnerable to excessive Ca^++^ influx [29], [65]–[67] while, the increased expression of a calmodulin fragment which normalizes intracellular Ca^++^ release improved motor function, weight loss and neuropathology in the R6/2 mouse model of HD [68]. In accordance with the inhibitory effect of opioids on Ca^++^ influx [64], we suggest that overexpression of striatal enkephalin *via* inhibition of Ca^++^ currents could also play a role in the improvement of behavioral dysfunction in the R6/2 mouse model of HD.

### Striatal pENK overexpression, density of enkephalin in striatal fibers and behavioral symptoms in R6/2 mice

Several studies revealed that altered activity of the GP is responsible for at least a part of the cognitive and motor symptoms of HD [69]–[71]. Indeed, endogenous metabolic marker, cytochrome oxidase (COX) histochemistry, showed an increased neuronal metabolic activity in the GP of transgenic rat model of HD [72]. Further studies also provide evidence that GP deep brain stimulation (DBS) has the potential to improve both motor and cognitive symptoms in transgenic rat model of HD [73], [74]. Within the GP both presynaptic and postsynaptic μ and δ opioid receptors are present [8]. Moreover, all GP projection neurons have local collateral axons [75] that may strongly inhibit GP neurons because they terminate on the perikarya and proximal dendrites of GP neurons [76]. As enkephalin expression is substantially reduced in the striatum and external pallidum of HD brain [25], we may also suggest that higher density of enkephalin positive fibers in GP of pENK-injected R6/2 mice, could maintain the inhibitory function of the GP through activation of presynaptic opioid receptors on collateral terminals and could then contribute to the improvement of behavioral symptoms in this group.

Interestingly, the density of enkephalin positive fibers in SN of pENK-injected R6/2 mice was significantly higher, two folds, than all other groups. Driving ectopic expression of enkephalin in a nucleus which normally does not receive enkephalinergic projections is not directly 'fixing' the neurochemical abnormality that is observed in HD. However, such ectopic expression allow us to propose that the increased level of enkephalin in SN of R6/2 mice having pENK overexpression, through activation of opioid receptors on striatonigral terminals may participate to the presynaptic inhibition of GABA–mediated transmission in substantia nigra reticulata (SNr) [77], [78] and be involved in the improvement of behavioral dysfunction, more especially abnormal clasping movement. Moreover, we may also suggest that in pENK-injected R6/2 mice, enkephalin-induced presynaptic activation of µ, δ and κ opioid receptors on subthalamic nucleus (STN) efferent [8], [79] by inhibition of excitatory neurotransmission in SNr might reduce the activity of SNr and could then contribute to disinhibition of thalamus and consequently to the improvement of behavioral symptoms in this group.

### Striatal pENK overexpression and striatal morphology

According to the particular vulnerability of striatal neurons to excessive Ca^++^ [29], [65]–[67], and the inhibitory effect of enkephalin on Ca^++^ influx [64], we expected that overexpression of striatal enkephalin *via* activation of opioid receptors and subsequent inhibition of Ca^++^ currents could also delay striatal neurons from degeneration in the R6/2 mouse model of HD. As the number of striatal neurons tended to be higher in pENK-injected mice compared to other groups of R6/2 and no difference was observed between pENK-injected R6/2 mice and WT, we may also propose that striatal pENK overexpression could delay striatal atrophy but could not prevent it at 10 weeks of age. However, striatal pENK overexpression had no beneficial effect on the decline in striatal volume of R6/2 mice. This can be explained in part by the atrophy of corticostriatal afferents which may contribute to the reduction of striatal volume. Indeed, the decline in striatal volume, starting as early as week 6, precedes striatal neuronal loss [30], parallel to the reduction of brain-derived neurotrophic factor (BDNF, trophic factor essential for neuronal survival) in different cortical areas from week 4 or 6 [30]. Therefore, we suggest that at early stage of the disease, striatal enkephalin could play a role in the reduction of excitotoxicity. Nevertheless, based on downregulation of transcription of several important neuronal genes [80]–[82] and further disease progression (atrophy of corticostriatal afferents and the progressive neurodegeneration of striatonigral dynorphin-containing neurons at later stage of disease), the level of opioid receptors might also be gradually downregulated, and the positive effect of enkephalin on striatal neuron morphology may eventually fail. Indeed, the reduction of striatal opioid receptor binding (20% to 40%) was shown in HD patients [83]. This proposition is in agreement with gradual decline in beneficial effect of striatal pENK overexpression on some behavioral symptoms in R6/2 from week 9 of age.

## Conclusion

We suggest that in HD, striatal enkephalin system at early stage of disease might play a role at attenuating illness symptoms *via*: a) the presynaptic inhibition of the corticostriatal glutamatergic input by opioid receptors activation, more specifically δORs; b) the inhibitory effect of opioid receptors on Ca^++^ influx; c) the reduction of cAMP signaling cascade.

The results of this study might open new horizon to investigate the cellular and molecular mechanisms by which enkephalin modulates motor response and signalling in HD, and may also contribute to the development of new therapeutical strategies and the gain in the quality of life in HD patients.
